# Richness and Composition of Niche-Assembled Viral Pathogen Communities

**DOI:** 10.1371/journal.pone.0055675

**Published:** 2013-02-26

**Authors:** Eric W. Seabloom, Elizabeth T. Borer, Christelle Lacroix, Charles E. Mitchell, Alison G. Power

**Affiliations:** 1 Department of Ecology, Evolution, and Behavior, University of Minnesota, St. Paul, Minnesota, United States of America; 2 Department of Biology, University of North Carolina, Chapel Hill, North Carolina, United States of America; 3 Department of Ecology and Evolutionary Biology, Cornell University, Ithaca, New York, United States of America; Centro de Investigación y de Estudios Avanzados, Mexico

## Abstract

The pathogen and parasite community that inhabits every free-living organism can control host vital rates including lifespan and reproductive output. To date, however, there have been few experiments examining pathogen community assembly replicated at large-enough spatial scales to inform our understanding of pathogen dynamics in natural systems. Pathogen community assembly may be driven by neutral stochastic colonization and extinction events or by niche differentiation that constrains pathogen distributions to particular environmental conditions, hosts, or vectors.

Here, we present results from a regionally-replicated experiment investigating the community of barley and cereal yellow dwarf viruses (B/CYDV's) in over 5000 experimentally planted individuals of six grass species along a 700 km latitudinal gradient along the Pacific coast of North America (USA) in response to experimentally manipulated nitrogen and phosphorus supplies. The composition of the virus community varied predictably among hosts and across nutrient-addition treatments, indicating niche differentiation among virus species. There were some concordant responses among the viral species. For example, the prevalence of most viral species increased consistently with perennial grass cover, leading to a 60% increase in the richness of the viral community within individual hosts (i.e., coinfection) in perennial-dominated plots. Furthermore, infection rates of the six host species in the field were highly correlated with vector preferences assessed in laboratory trials. Our results reveal the importance of niche differentiation in structuring virus assemblages. Virus species distributions reflected a combination of local host community composition, host species-specific vector preferences, and virus responses to host nutrition. In addition, our results suggest that heterogeneity among host species in their capacity to attract vectors or support pathogens between growing seasons can lead to positive covariation among virus species.

## Introduction

Historically, the study of host-pathogen interactions has focused on three components essential to the completion of a pathogen lifecycle forming the ‘disease triangle’: an infectious microbe, a host, and a favorable environment [1], [2], [3]. While many studies have probed the interactions among the vertices of the disease triangle (e.g., [1], [2], [3], [4], [5], [6], [7], [8], [9], [10], [11], [12], [13], [14], [15], [16]), investigations of the larger ecological context in which host-pathogen-environment interactions occur, including the interactions among multiple microbial species infecting the same host, multiple species in a host community, and the impact of multiple abiotic parameters have generally been observational or limited in spatial scope [17], [18], [19], [20], [21].

Nevertheless, the experimental investigation of interacting pathogen communities is important, because every free-living organism is host to a wide array of microbial symbionts that range in effects from pathogenic to mutualistic [22], [23], [24], [25], [26], [27], and the richness and composition of pathogen and parasite communities in particular can alter nearly every vital rate of a host including lifespan and reproductive output [27], [28], [29], [30], [31], [32], [33]. For example, coinfection by multiple pathogens can increase the severity of disease, as is the case for humans infected by multiple strains of human immunodeficiency virus (HIV), HIV and malaria, or the hepatitis C virus and the trematode *Schistosoma mansonii* [30], [31], [32], [33]. Infection by multiple viral or fungal pathogens can also increase mortality in plant hosts [28], [29]. Despite the importance of interactions among pathogens within a host, we know relatively little about the assembly of pathogen communities in natural systems, and we know of no field experiments that have been replicated at scales large enough to inform our understanding of how the biotic and abiotic context interact to mediate the natural assembly of pathogen communities in multi-host systems.

As in the community assembly of free-living species, variation in pathogen community structure can arise from stochastic infection events (i.e., neutral or dispersal assembled communities) or from niche differentiation that constrains the pathogens present in each host (i.e., niche assembled communities) [25], [34], [35]. Although neutral and niche processes are not mutually exclusive, their expectations can be used to assess the importance of species' traits in controlling community composition, beyond the variation expected from neutral processes [36], [37]. In particular, neutral theory predicts that species are functionally equivalent and should show no consistent changes in relative abundance across changing environments, including predictable composition among host species, sites, or along fertility gradients [36], [37]. In contrast, predictable variation in species composition across environmental gradients suggests trait-based sorting, supporting the importance of niches for understanding pathogen coexistence [36], [37]. Understanding the relative contribution of neutral and niche processes for coinfection ultimately could inform strategies for disease management and prevention.

Pathogen niches can be defined by processes that alter host, pathogen, and vector interactions [2], [4], [5], [6], [7], [8]. These processes can operate at a wide range of scales, from regional gradients in abiotic conditions such as climate or nutrient supply [17], [21], [25], [38], [39], [40] or biotic host and vector community composition [19], [20], [25], [40], [41], [42] down to pathogen and vector interactions with individual hosts [9], [10], [11], [43], [44], [45]. Although the abiotic and biotic environment has been shown to regulate many pathogens of global importance based on observational studies (e.g., malaria, dengue fever, Lyme disease, and West Nile virus) [2], [4], [5], [17], [19], [21], [39], [41], [43], experimental work has been largely confined to plant systems due the ethical and logistical difficulty of conducting large-scale manipulations of human and wildlife pathogens [40], [46], [47], [48].

Here we measure the prevalence and co-occurrence of five viral pathogens (barley and cereal yellow dwarf viruses; B/CYDV's) in over 5000 experimentally planted hosts of six different species at five sites spanning a 700 km latitudinal gradient along the Pacific coast of North America (USA). At each site, we experimentally manipulated supplies of nitrogen and phosphorus, creating within-site gradients in fertility and stoichiometry. Using data on regional viral occurrence and coinfection within hosts by these five viruses, we assess the extent to which the composition of viral species within hosts changed among host species, natural gradients in local plant composition, sites, and local gradients of two different nutrients. We use these data to quantify the processes and contingencies of pathogen community assembly in a natural environment.

## Methods

### Study System

B/CYDVs are aphid-vectored RNA viruses (family Luteoviridae) that infect the phloem of grasses and cause one of the most devastating of all diseases in cereal crops worldwide [49], [50]. In addition, B/CYDVs have been implicated as a causal agent in one of the most dramatic and persistent biological invasions worldwide, the conversion of nine million ha of grasslands in California (USA) from native perennial to exotic annual dominated [51], [52], [53]. B/CYDVs are known to infect over 150 grass species and are carried by over 25 aphid species [50], [54]. There is no vertical transmission of the viruses to the seeds (hosts only become infected through vector feeding after germination), and the viruses do not replicate within the aphid vectors [3], [55]. Hosts become infected through vector feeding after germination and the viruses spread systemically through the host phloem tissue. Once infected, perennial grasses are not known to clear themselves of viruses and thus generally accumulate viruses over time [56]. Because seeds do not carry B/CYDV infections, grasses with an annual life history are uninfected at the start of each growing season.

BYDVs and CYDVs belong to various viral genera [28].We track five common species (BYDV-MAV, BYDV-PAV, BYDV-SGV, BYDV-RMV, CYDV-RPV). The most common aphid vectors at our study sites are *Rhopalosiphum padi*, *R. maidis*, *Sitobion avenae*, *Metopolophium dirhodum*, and *Schizaphis graminum* (E. T. Borer, unpublished data), and these vector species differ strongly in their transmission efficiency of each virus species. Three of the aphids are generalist vectors that can transmit two viruses. *R. padi* is effective at transmitting BYDV-PAV and CYDV-RPV and *S. avenae* and *M. dirhodum* are effective at transmitting BYDV-MAV and BYDV-PAV [28]. Two aphid species, *S. graminum* and *R. maidis*, are specialists and can only transmit a single virus effectively (BYDV-SGV and BYDV-RMV, respectively) [28].

### Experimental Design

#### Field nutrient-addition experiment

We conducted a full factorial experiment of nitrogen and phosphorus addition at five sites in California (McLaughlin Reserve and the Sierra Foothill and Hopland Research and Extension Centers) and Oregon (Baskett Slough and William Finley National Wildlife Refuges). Borer et al. [40] provide additional details on study sites and experimental design. The California sites were arrayed in longitudinal transect at approximately 39°N latitude and represent a gradient in mean rainfall from 939 mm yr^−1^ near the coast (Hopland) to 711 mm yr^−1^ further inland (Sierra Foothill). The two Oregon sites (Baskett and Finley) were at about 45°N latitude and have an average of 1198 and 1030 mm of rain per year, respectively. In addition to rainfall, the latitudinal gradient also corresponds to increasing cover of perennial grasses [25].

We established two experimental blocks at each site, with each block composed of four 40×40 m plots. These plots were randomly assigned to one of the four factorial combinations of phosphorus addition (Control or 4.3 g P m^−2^ yr^−1^ as triple super phosphate) or nitrogen addition (Control or 4 g N m^−2^ yr^−1^ as calcium nitrate). Fertilization treatments were applied quarterly starting in December 2006. In summary, the entire experiment was composed of 40 experimental units (40×40 m plots)−5 sites×2 blocks×2 levels of nitrogen×2 levels of phosphorus. Due to a field error, all samples from 5 plots at the Finley site could not be assayed for B/CYDVs.

We selected six target host species that are common throughout Pacific Coast grasslands and represent congeneric or contribal pairings of a native perennial and exotic annual grass. There are few native annual grasses in these systems [57]. The phylogenetic groupings were as follows (annual/perennial): Brome (*Bromus hordeaceus*/*Bromus carinatus*), Oat (*Avena fatua/Koeleria macrantha*), and Rye (*Taeniatherum caput-medusae/Elymus glaucus*). Note that *K. macrantha* is referred to by its synonym *K. cristata* by Borer et al. [40] in their related analysis of data from this experiment. Seeds of each species were collected at each site or from as nearby as possible. Hosts were pre-germinated in 25×25 mm soil plugs in greenhouses at Oregon State University, Corvallis, Oregon and transplanted to each site in January 2008, prior to the first aphid flight. One individual of each species was planted within a series of 20–30 quadrats (20×50 cm) arrayed along transects within each experimental plot. Except for being marked with twist-ties, the experimental hosts rapidly became indistinguishable from naturally recruiting individuals in the community.

We collected all aboveground tissue from the 5,095 surviving experimental hosts in late May and early June 2008, after about 5 months of growing in the field. These aboveground tissues were weighed to the nearest 0.01 g and assayed for five B/CYDV species (BYDV-MAV, BYDV-PAV, BYDV-SGV, BYDV-RMV, CYDV-RPV) using double antibody sandwich enzyme-linked immunosorbent assay (DAS-ELISA) with antibodies supplied by Agdia, Elkhart, IN, USA. Individual hosts were randomly distributed between the Mitchell lab at UNC and the Power lab at Cornell University. In rare cases of putative infection by serologically related viruses that were associated nearly 1∶1 within hosts, we regarded the weaker of the two reactions as a cross-reaction to the more reactive virus rather than a coinfection. This conservative approach did not affect the overall prevalence estimates but may have somewhat reduced our estimate of coinfection by serologically related viruses. Note that we were only able to assay about half of the hosts for BYDV-SGV (2,320 hosts) due to a lack of antibody. We conducted analyses on both subsets of the data (5,095 hosts with 4 viruses and 2,320 hosts with 5 viruses). As results are qualitatively similar, we present the results with the full suite of viruses but fewer individual hosts.

We collected data on plant biomass and community composition at two locations along transects in each of the 40×40 m plots. We estimated total plant biomass in each plot by clipping all aboveground plant material in two 0.1×1 m strips, sorting it into categories (previous year's dead material and current year's grass, forb, legume, woody, bryophyte growth), drying it to constant mass at 60°C, and weighing it to the nearest 0.01 g. We estimated areal cover of all species in an adjacent 0.5×1 m quadrat. Cover of each species was estimated independently, so that total summed cover exceeds 100% in quadrats with multilayer canopies.

#### Laboratory aphid experiments

We use aphid fecundity and preference data from two experiments published by Borer et al. [58] to explain prevalence differences among host species: *Preferential herbivory* and *Greenhouse multivector fecundity* (Experiments III and IV in Borer et al. 2009a). In the *Preferential-herbivory experiment* 30 aphids (*R. padi*) were placed in the center of 3.78 l L pots in which one individual of 10 different host species (including our six focal species) were planted around the pot perimeter. Aphids were counted on all plants after 24 hours and, because plants of the same age differed in size, counts were calculated on a per gram of host tissue basis. For the *Greenhouse multivector fecundity experiment*, three species of aphids were placed in small mesh enclosures on replicate individuals of six our focal host species that were subjected to two levels of nitrogen (control or nitrogen addition). Our measure of fecundity was the number of offspring produced by each adult during a four day assay. See Borer et al. [58] for additional details of the aphid studies.

#### Statistical analyses

All analyses were conducted using R version 2.12 [59]. We tested for overall changes in the viral community using Permutational Multivariate Analysis of Variance (PerMANOVA) using the adonis function in the R vegan library. PerMANOVA is analysis of variance that compares distance matrices in which significance is determined using a permutation test. PerMANOVA is analogous to MANOVA and redundancy analyses [60], [61], [62]. Here we used 999 permutations and Jaccard's distance matrices. Only infected plants were included in the analyses, as uninfected plants all had a distance of zero and were uninformative. In the PerMANOVA tests, we use two approaches to control for spatial variability in our tests for the effects of the experimental treatments applied at the plot scale (nitrogen, phosphorus, and host species). First, we constrain the permutations to only occur among experimental units within each block (blocks are treated as strata in the adonis function). Second, we include key covariates (e.g., host community composition) while allowing permutations to be unconstrained. The first method of constrained permutations is the most powerful test of our experimental treatments, while the second method provides more biological insight into larger scale variability. Univariate analyses used mixed-effects regression models using the nlme R library. State, site and block within site were included as random effects in all mixed models. Fixed effects were determined using backwards selection as in Crawley [63].

## Results

Overall infection prevalence across all 2320 hosts assayed for all five viruses was 25.5%, and 44% of the infected hosts were coinfected by multiple viruses. Infection patterns were similar in the larger data set (5095 hosts) assayed for four viruses; infection was 23.6% and coinfection of infected plants was 52.5%. There was a great deal of variation among the plots in terms of overall virus prevalence (0–68%), plant biomass (46–481 g m^−2^), annual grass cover (0–149%), perennial grass cover (0–66%), forb (non-host) cover (0–105%) and host (grass) species richness (1–7.2 species m^−2^). See supplement in Borer et al. [40] for a table of site-level means of total infection prevalence, soil chemistry, plant biomass, and plant species cover.

Host survival ranged from 79–88% among the host species, but was not statistically different [40]. Prevalence of each virus species across the study plants were as follows: BYDV-RMV (14.0%), BYDV-SGV (12.4%), BYDV-PAV (11.1%), CYDV-RPV (9.5%), and BYDV-MAV (9.4%). The mean number of viruses carried by infected hosts was 2.26±0.08 SEM. As has been found in other data sets [25], [56], viruses carried by the same vectors were strongly correlated with one another in coinfected hosts. Among the 10 pairwise combinations of virus species, the highest correlations were between the *S. avenae* vectored viruses, BYDV-MAV and BYDV-PAV, (r = 0.56, p<0.001) and the *R. padi* vectored viruses, BYDV-PAV and CYDV-RPV, (r = 0.43, p<0.001).

**Figure 1:**
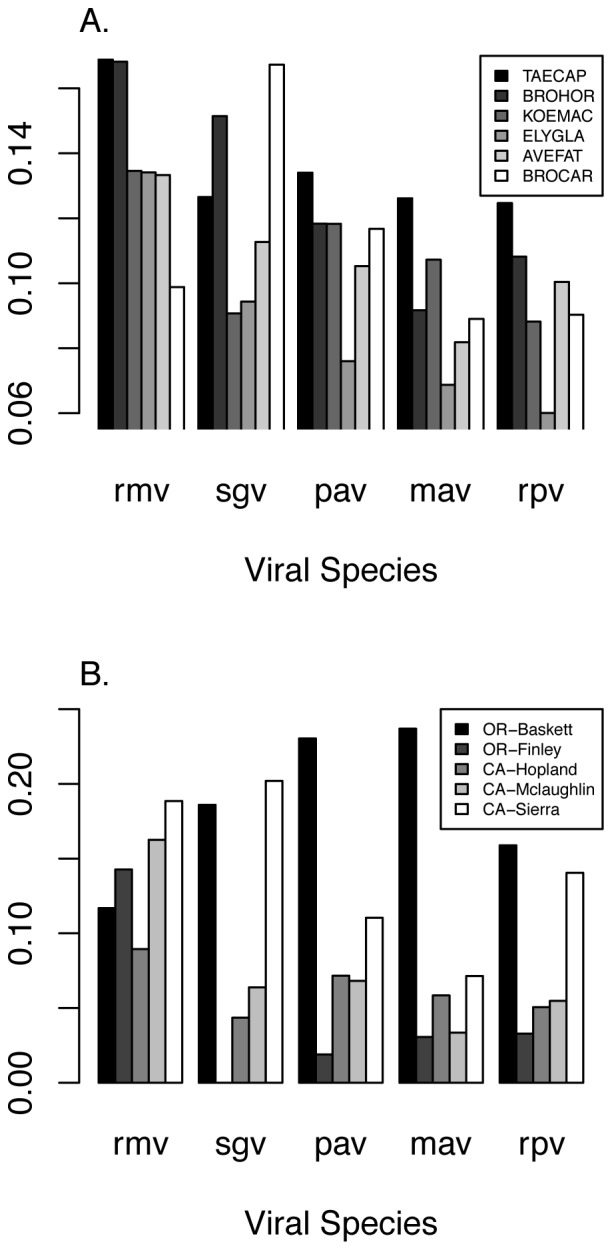
Prevalence of five plant viruses in (A) six grass hosts: *Avena fatua* (AVEFAT), *Bromus carinatus* (BROCAR), *Bromus hordeaceus* (BROHOR), *Elymus glaucus* (ELYGLA), *Koeleria macrantha* (KOEMAC), and *Taeniatherum caput-medusae* (TAECAP), and (B) at five study sites in Oregon and California.

Among naturally infected hosts in control plots, the viral community varied at all spatial scales greater than individual plots (i.e. >1600 m^2^: block, site and state; Table S1). Furthermore, viral community composition differed among hosts in models that controlled for among-plot variability by constraining permutations to occur within plots (e.g., among quadrats within a block; Table S2; Figure 1). In particular, *T. caput-medusae* had consistently higher virus infection prevalence than other species, and *A. fatua*, generally considered a reservoir species for these pathogens [47], [52], had low to intermediate infection prevalence by all viral groups compared to other host species (Table S3; Figure 1).

**Figure 2:**
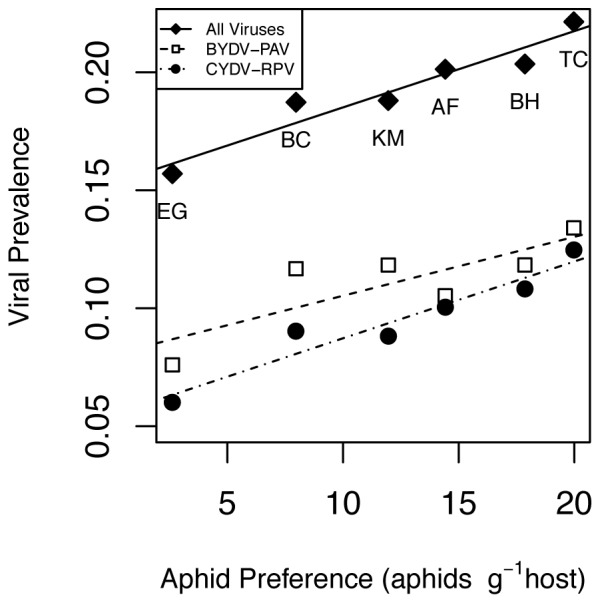
Correlations of an aphid vector (*Rhopalosiphum padi*)'s feeding preference with the B/CYDV prevalence. Plot show overall B/CYDV prevalence (infection by any virus) and the prevalences of two viruses (BYDV-PAV and CYDV-RPV) vectored by *R. padi*. The six grass hosts are as follows: *Avena fatua* (AF), *Bromus carinatus* (BC), *Bromus hordeaceus* (BH), *Elymus glaucus* (EG), *Koeleria macrantha* (KM), and *Taeniatherum caput-medusae* (TC). Correlations between feeding preference and overall prevalence of infection (r = 0.96), BYDV-PAV (r = 0.82), and CYDV-RPV (r = 0.96) were significantly greater than 0 (p<0.05).

While viral community composition differed among host species, these among-host differences were not associated with host traits (p>0.05) including host lifespan (annual or perennial), provenance (native or exotic), or phylogenetic group (bromes, oats, or rye), and so we only present models here where species identity is treated as a single categorical variable. The strongest covariate explaining the relative prevalence rates in the different host species was the preference by the aphid vector, *R. padi*, for those host species in the laboratory Preferential-herbivory experiment) (Figure 2). There were no significant relationships between prevalence of the associated viruses in field hosts and aphid fecundity on the same host species in the laboratory Greenhouse multivector fecundity experiment (p>0.05).

**Figure 3:**
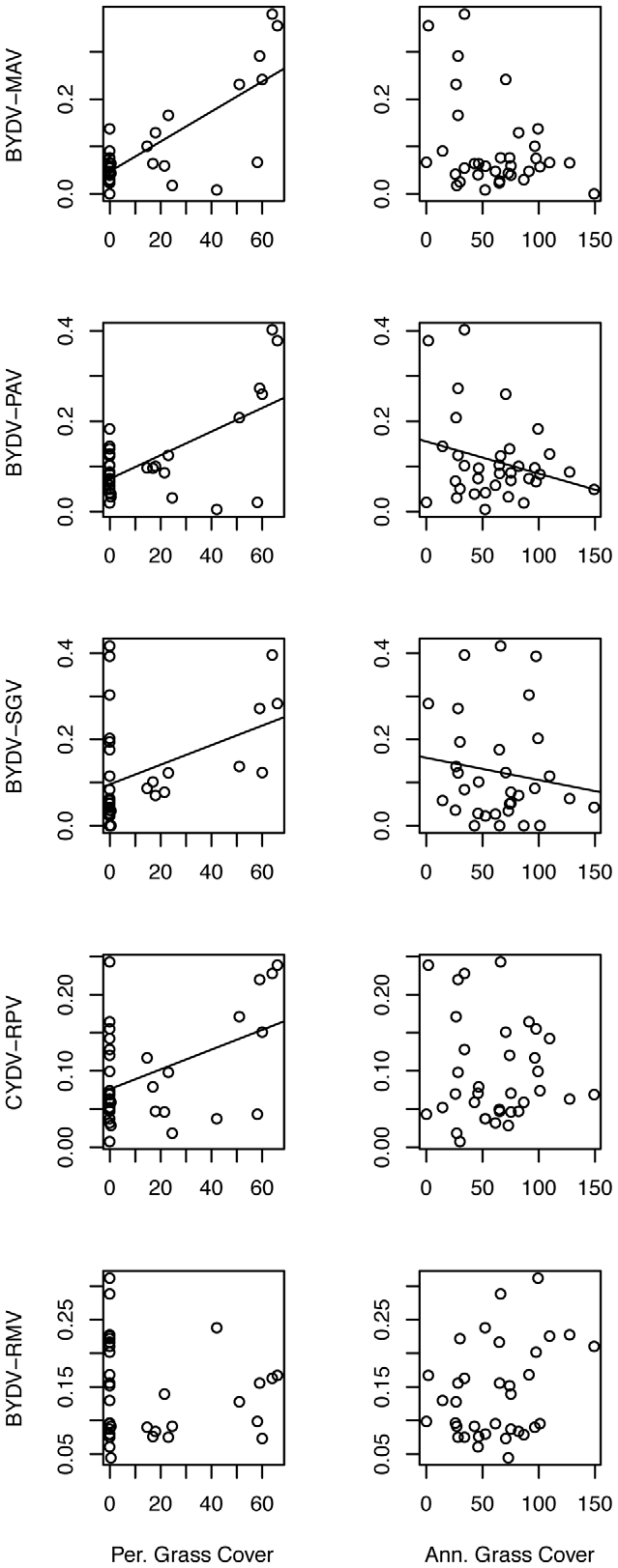
Prevalence of five viruses along gradients in plot host community composition. Lines indicate the slope of significant relationships.

**Figure 4:**
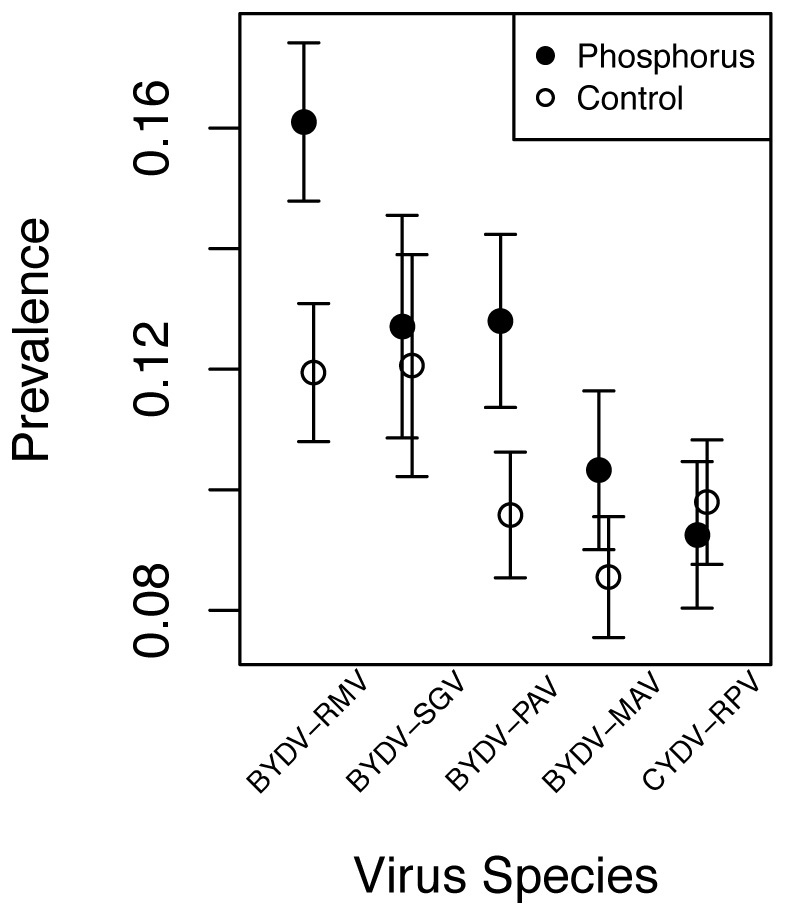
Effects of phosphorus addition on the prevalence of five viral species. Error bars represent ± one SE from the mean.

Viral community composition also was correlated with host community composition (cover of perennial grasses, annual grasses and forbs), aboveground plant biomass, and phosphorus addition (Tables S4 and S5; Figures 1, 3, 4). Among-host differences in viral community composition were not significant indicating that they were obscured by large-scale environmental gradients (Table S4).

**Figure 5:**
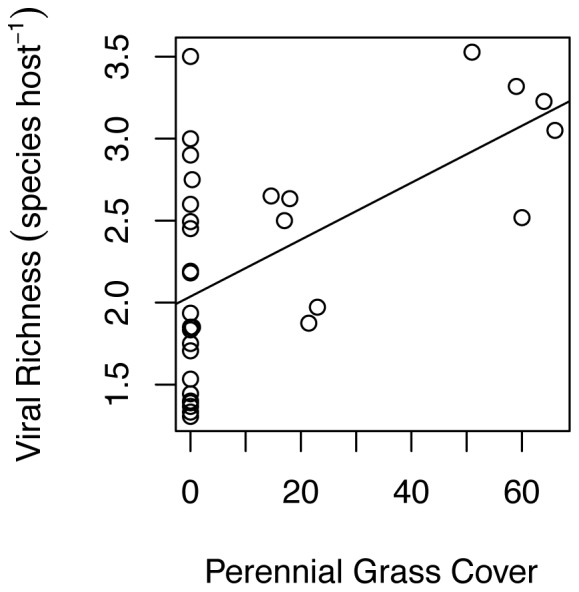
Coinfection (viral species richness per infected host) of five viruses along a gradient of perennial grass cover. The line represents the slope of this relationship.

To understand the nature of these strong effects of host-community composition, phosphorus, and host identity on viral community composition, we examined the univariate coefficients for the responses of individual viruses in the PerMANOVA models (Table S5). All viruses except BYDV-RMV increased with cover of perennial grasses, and BYDV-PAV and BYDV-SGV decreased with cover of annual grasses (Figure 3). As a result of this concordant response, viral richness within infected hosts increased 58% from 1.97 virus species per host in annual-only plots to 3.13 virus species per host in plots with more than 50% cover of perennial grasses (Figure 5; Table S6). Two viruses, BYDV-RMV and BYDV-PAV, increased in prevalence by approximately 30% in response to phosphorus addition, while the remaining viruses were unaffected (Figure 4).

## Discussion

Much of our understanding of host-pathogen interactions arises from detailed studies examining the coupled interactions between a single pathogen, one host species, and the environment [3], [6], [7], [8]. It is increasingly apparent that interactions in the disease triangle are mediated by a larger ecological context that includes multiple pathogens and host species, gradients of abiotic parameters, and complex landscapes [5], [19], [21], [41], [43], [64], [65]. Nevertheless, few studies have attempted to examine the drivers of multi-pathogen communities across a range of hosts and environmental conditions.

In a unique, regionally replicated, multi-host experiment, Borer et al. [40] found that that the infection of hosts by B/CYD viruses increased with additional phosphorus and the cover of long-lived, perennial hosts. While this host-centered analysis clarified the role of these factors for host infection, it did not clarify the effects of environmental factors on the composition and richness of the virus community within hosts. Here we show how changes in infection prevalence at the scale of hosts are determined by the niches of individual virus species. For example, our current analyses demonstrate that the increase in host infection prevalence with increasing perennial host abundance was caused by a concordant response of most viral species leading to a corresponding increase in viral richness within hosts. In contrast, the increase in overall prevalence arising from phosphorus addition [40] was primarily driven by an increase in two viruses (BYDV-PAV and BYDV-RMV). Thus, our current virus-centered analysis of environmental drivers of virus community diversity and composition within hosts provides novel insights into mechanisms leading to patterns of host infection.

Our current work demonstrates that the composition of the viral community within individual hosts varied across a wide range of spatial scales from hundreds of km to a few meters. Among control plots, representing natural biotic and abiotic gradients, the composition of the viral community differed among states (Oregon or California), sites, and blocks within sites. Specifically, the viral community varied with local host-community composition, phosphorus addition, host species identity, and vector foraging preferences among host species, suggesting the greater importance of niche differentiation compared to neutral processes in determining virus community assembly within hosts.

Host community composition was the most important determinant of spatial variability in viral community richness and composition, and these host-specific patterns of virus prevalence could be the result of interspecific variation in plant susceptibility to virus infection. Four of the five viruses increased in prevalence as perennial grass cover increased, resulting in a net 60% increase in the mean number of virus species in each host. Virus responses to host identity and nutrient supply were more complex, with the prevalence of each virus bearing a unique signal of response to phosphorus addition and host identity. For example, phosphorus addition had the strongest effects on the prevalence of BYDV-PAV and BYDV-RMV, and viruses differed in their ordering of the host species in terms of prevalence. Viruses depend on host cellular machinery to complete their replication cycle and require specific interactions between virus- and host-encoded factors [15]. A disruption of virus-host recognition, which could be caused by the presence of a tolerance or resistance gene in the host genome, can inhibit viral infection [66]. While some tolerance and resistance sources have been identified in wild relatives of cultivated grasses [67], [68], [69], these have been very limited in number. Our results suggest that their presence in the six host species selected in this study and impact on virus prevalence would be a fruitful avenue for future investigation.

In addition to host identity and phosphorus addition, virus community composition also was related strongly to differences in vector competence and host preference. As has been seen in observational studies and modeling work in this system [25], [56], viruses that share a vector coinfect hosts more frequently. In addition, virus distribution among hosts reflected vector-feeding preferences. Host specificity for the viruses carried by the widespread and common vector, *R. padi*, (BYDV-PAV and CYDV-RPV) were predicted extremely well by aphid preferences among host species in lab experiments (r = 0.82 and r = 0.96 respectively), and this correlation with vector preference was reflected in the overall prevalence of these viruses in the field, demonstrating that vector behavior and choices are important components of the non-random viral community assembly observed in this system.

Recent research in disease ecology has demonstrated that abiotic and biotic contexts are critical determinants of pathogen dynamics. A particular focus has been the prediction of human impacts, such as nutrient addition, habitat loss, biological invasions, and climate change, on pathogen epidemics [21], [38], [40], [41], [51], [70]. For example, the impacts of such globally critical human diseases as malaria and Lyme disease are mediated by both climate and the composition of the host community [39], [41], [43]. Recent work on plant pathogens has led to major advances by experimentally testing ecological drivers of pathogen distribution at scales or in ways that are logistically or ethically impossible in human and wildlife pathogen systems [40], [46], [47], [48], [71]. The work here extends our knowledge of the more complex dynamics of multi-host, multi-pathogen systems.

The importance of linking host and pathogen communities is highlighted by our finding that multiple viruses coinfected about half of infected hosts, and that coinfection rates arise from predictable differences in the environmental niches of the pathogens comprising the community. Specifically, we have demonstrated experimentally that the structure of multi-pathogen communities arises from niche differentiation among individual pathogens in response to the abiotic environment, host community composition, and the characteristics of individual hosts. For example, while phosphorus supplies increased overall infection rates [40], our current analyses demonstrate that the aggregate response predominantly reflects the large increases of a few virus species (BYDV-PAV and BYDV-RMV). Thus, the hosts in a phosphorus-rich system have both greater total infection prevalence and altered pathogen composition, contrasting strongly with the expectation that changing environments should not lead to alterations of species richness for neutrally-assembled communities [36], [37]. The altered community composition can be important, as infection by multiple viruses can reduce host fitness, especially if viruses are distantly related [28], [56].

Nutrient addition can act on pathogens through multiple pathways such as alteration of host community composition, host quality for vectors, pathogen reproduction, and host tolerance of infection. For example, nutrient additions often lead to declines in plant species diversity [79], [80], and declines in species diversity have been shown to lead to increased pathogen prevalence via the dilution effect [19], [20], [41], [48]. In this experiment, phosphorus addition increased total host biomass and total plant biomass, however it did not alter species richness or the relative abundance of perennial grasses [40]. We controlled for changes in total productivity and community composition in our analyses, and still found strong effects of phosphorus on the prevalence of some virus species (BYDV-PAV and BYDV-RMV). Thus, it is unlikely that the phosphorus effects on infection were driven solely by changes in vector or plant community composition, diversity, or productivity. While fertilization can alter vector performance or preference, aphid vector responses are primarily driven by nitrogen and not phosphorus [58]. Further, in experimental inoculations of our six focal host species, hosts physiologically adapted to greater nutrient supplies, e.g. with greater leaf nitrogen concentrations, were more susceptible to infection by BYDV-PAV [81]. Phosphorus has been shown to control viral reproduction rates [76], but the role of nutrient limitation of growth for each virus in this system remains unresolved and is a fruitful avenue for future research. Similarly, we do not have direct measurements of whether nutrient supplies differentially alter host tolerance or resistance to each of the B/CYDV's, as is the case for some other pathogens [77], [78]. Despite the complexity of these interactions, the consistently higher pathogen loads of hosts in eutrophic systems have important implications for disease management. Human activity has led to an eightfold increase in phosphorus and fourfold increase in nitrogen in the earth's ecosystems [72], [73], [74], [75], and nutrient additions have been shown to alter pathogen prevalence or reproduction in a wide array of human, animal, and plant systems [21], [40], [76], [77], [78].

Infection rates by each virus species generally increased with phosphorus addition, but the magnitude of this increase differed among host species. In particular, *Avena fatua*, an exotic annual grass that is known as a reservoir host species in this and other grass systems [47], [53], was associated with the greatest increase in prevalence of 4 of the 5 viral species in response to additional nutrients. In contrast, virus species responses to fertilization in *Elymus glaucus*, a widespread native perennial, consistently were less pronounced compared to *A. fatua*. More generally, these results highlight the important role of host species identity for determining virus infection and coinfection rates. Variation in host species traits is important for determining transmission rates and patterns of infection in many generalist pathogens including sudden oak death, Lyme disease, and West Nile virus [41], [81], [82], [83], and our current results suggest that host nutrition and physiology also may interact to control coinfection rates by related pathogen species.

Recent research in disease ecology has been building linkages between host community composition, host diversity and infection risk for single pathogens or specialist pathogens (e.g., pathogen spillover and the dilution effect) [19], [20], [41], [47], [48]. While there were no effects of host species richness in our system, we did find that infection risk by most viruses increased with the presence of long-lived perennial hosts as has been shown for aggregate infection risk [40]. Along a gradient from annual-dominated to perennial-dominated host communities, we found that the aggregated response of the individual viruses led to a 60% increase in the number of viruses carried by individual hosts. Given that coinfection rates can have strong effects on pathogen transmission and virulence [84], [85], [86], [87], it is important to expand the investigation of host community effects of pathogens to include coinfection.

Finally, we found that host-vector interactions were critical determinants of infection risk, as has been found in other systems such as mosquito-vectored pathogens [11], demonstrating that vector preference can provide a general key to predicting host infection rates in both animals and plants. Foraging preference of aphid vectors in the lab was a surprisingly strong predictor of infection among hosts in the field. The role of vector behavior is particularly important, as vector transmitted pathogens are the dominant form of emerging plant and animal diseases [88], [89]. There have been attempts to exploit vector feeding preferences to benefit human health by maintaining preferred hosts in close proximity to humans (i.e., zooprophylaxis) [43], [90], [91], however preferred hosts may also increase vector densities so it remains unclear if host preference will alter infection risk over longer periods of time [43]. Our results suggest that vector foraging preferences led to the observed strong and consistent differences in infection risk among hosts grown in close proximity. This linkage between laboratory measurements of vector behavior and infection risk across large geographic gradients suggests that disease control strategies may be able to effectively exploit vector preference to control disease risk.

While there has been growing evidence that host community and ecosystem context are critical drivers of host-pathogen interactions [19], [20], [21], [40], [41], [47], [48], [76], [77], [78], most of the existing evidence is based on observational studies, is not replicated at regional scales, or has focused on single pathogens. Here we experimentally demonstrate pathogen niche differentiation arising from differential responses of each pathogen species to host identity, the suite of hosts in the surrounding community, and the supply of nutrient resources. We also found concordant responses across these coexisting pathogens; most viral species were more common in the presence of long-lived hosts. This concordant response resulted in increases in both aggregate viral prevalence and coinfection within hosts. The net result was a mosaic of coinfection patterns in which the pathogen load of each host bore the imprint of its own identity and its biotic and abiotic context. Additional regionally-replicated, experimental studies of coinfection among other pathogen groups will provide the more general framework necessary to forecast the effects of human-induced changes to climate and nutrient supplies on global patterns of disease [46].

## Supporting Information

Table S1 Results of permutational multivariate analysis of variance (PERMANOVA) testing similarity in the viral community (BYDV-MAV, BYDV-PAV, BYDV-SGV, BYDV-RMV, CYDV-RPV) in infected individuals of six grass species (*Avena fatua*, *Bromus carinatus*, *Bromus hordeaceus*, *Elymus glaucus*, *Koeleria macrantha*, and *Taeniatherum caput-medusae* among states (Oregon or California), sites within states, blocks within sites, quadrats within plots, and host species within a quadrat. All terms not in the reduced model presented here were not significant (p>0.05). Sum of squares are sequential and so represent the nested spatial structure. Note these data are only from control plots and so represent background variability in the viral community. In addition, there is only a single plot per block, so there is not estimate of variability among plots within blocks.
(DOCX)

Table S2 Results of permutational multivariate analysis of variance (PERMANOVA) testing the effect of factorial additions of nitrogen and phosphorus on the prevalence of five different viruses (BYDV-MAV, BYDV-PAV, BYDV-SGV, BYDV-RMV, CYDV-RPV) in infected individuals of six grass hosts (*Avena fatua*, *Bromus carinatus*, *Bromus hordeaceus*, *Elymus glaucus*, *Koeleria macrantha*, and *Taeniatherum caput-medusae*. Permutations were constrained within unique block by site combinations testing for the effects of nitrogen, phosphorus, and host species after controlling for variation among blocks, sites, and states. Full model contained all two-way interactions between nitrogen, phosphorus, and host species. Note that among-host differences were not associated with host lifespan (annual or perennial), provenance (native or exotic), or phylogenetic group (bromes, oats, or rye).
(DOCX)

Table S3 Results of permutational multivariate analysis of variance (PERMANOVA) testng the effect of perennial grass cover, annual grass cover, forb cover, and factorial additions of nitrogen and phosphorus on the prevalence of five different viruses (BYDV-MAV, BYDV-PAV, BYDV-SGV, BYDV-RMV, CYDV-RPV) in infected individuals of six grass species (*Avena fatua*, *Bromus carinatus*, *Bromus hordeaceus*, *Elymus glaucus*, *Koeleria macrantha*, and *Taeniatherum caput-medusae*). Full model contained total live biomass, host species richness, perennial grass cover, annual grass cover, forb cover and all two-way interactions between.
(DOCX)

Table S4 Linear coefficients for individual viral species from PERMANOVA (Table S2) testing the effect of factorial additions of nitrogen and phosphorus on the prevalence of five different viruses (BYDV-MAV, BYDV-PAV, BYDV-SGV, BYDV-RMV, CYDV-RPV) in infected individuals of six grass species (*Avena fatua*, *Bromus carinatus*, *Bromus hordeaceus*, *Elymus glaucus*, *Koeleria macrantha*, and *Taeniatherum caput-medusae*).
(DOCX)

Table S5 Linear coefficients for individual viral species from PERMANOVA (Table S4) testing the effect of perennial grass cover, annual grass cover, forb cover, and factorial additions of nitrogen and phosphorus on the prevalence of five different viruses (BYDV-MAV, BYDV-PAV, BYDV-SGV, BYDV-RMV, CYDV-RPV) in infected individuals of six grass species (*Avena fatua*, *Bromus carinatus*, *Bromus hordeaceus*, *Elymus glaucus*, *Koeleria macrantha*, and *Taeniatherum caput-medusae*).
(DOCX)

Table S6 Results of mixed-effects model testing the effect of perennial grass cover, annual grass cover, forb cover, and factorial additions of nitrogen and phosphorus on viral species richness (coinfection) by five different viruses (BYDV-MAV, BYDV-PAV, BYDV-SGV, BYDV-RMV, CYDV-RPV) in infected individuals of six grass hosts (*Avena fatua*, *Bromus carinatus*, *Bromus hordeaceus*, *Elymus glaucus*, *Koeleria macrantha*, and *Taeniatherum caput-medusae*. Full model contained total live biomass, host species richness, perennial grass cover, annual grass cover, forb cover and all two-way interactions between nitrogen, phosphorus, and host species. State, Site, Block, and Plot were treated as nested random effects.
(DOCX)
