# Supplementary material for: Richness and Composition of Niche-Assembled Viral Pathogen Communities
Source: PLoS One. 2013 Feb 26;8(2):e55675. doi: 10.1371/journal.pone.0055675 (PMC3582609; doi:10.1371/journal.pone.0055675)
Supplement: Table S1 — Results of permutational multivariate analysis of variance (PERMANOVA) testing similarity in the viral community (BYDV-MAV, BYDV-PAV, BYDV-SGV, BYDV-RMV, CYDV-RPV) in infected individuals of six grass species (Avena fatua, Bromus carinatus, Bromus hordeaceus, Elymus glaucus, Koeleria macrantha, and Taeniatherum caput-medusae among states (Oregon or California), sites within states, blocks within sites, quadrats within plots, and host species within a quadrat. All terms not in the reduced model presented here were not significant (p>0.05). Sum of squares are sequential and so represent the nested spatial structure. Note these data are only from control plots and so represent background variability in the viral community. In addition, there is only a single plot per block, so there is not estimate of variability among plots within blocks. (DOCX) [file pone.0055675.s001.docx]

**Table S1.** Results of permutational multivariate analysis of variance (PERMANOVA) testing similarity in the viral community (BYDV-MAV, BYDV-PAV, BYDV-SGV, BYDV-RMV, CYDV-RPV) in infected individuals of six grass species (*Avena fatua*, *Bromus carinatus*, *Bromus hordeaceus*, *Elymus glaucus* , *Koeleria macrantha*, and *Taeniatherum caput-medusae* among states (Oregon or California), sites within states, blocks within sites, quadrats within plots, and host species within a quadrat. All terms not in the reduced model presented here were not significant (p > 0.05). Sum of squares are sequential and so represent the nested spatial structure. Note these data are only from control plots and so represent background variability in the viral community. In addition, there is only a single plot per block, so there is not estimate of variability among plots within blocks.

| Source | D.F. | S.S | M.S. | F | p |
| --- | --- | --- | --- | --- | --- |
| State | 1 | 3.409 | 3.409 | 16.584 | 0.001 |
| Site | 2 | 1.170 | 0.585 | 2.846 | 0.013 |
| Block | 4 | 1.567 | 0.392 | 1.906 | 0.031 |
| Residuals | 115 | 23.642 | 0.206 | 0.794 |  |
| Total | 122 | 29.789 | 1.000 |  |  |
