# Supplementary material for: Richness and Composition of Niche-Assembled Viral Pathogen Communities
Source: PLoS One. 2013 Feb 26;8(2):e55675. doi: 10.1371/journal.pone.0055675 (PMC3582609; doi:10.1371/journal.pone.0055675)
Supplement: Table S2 — Results of permutational multivariate analysis of variance (PERMANOVA) testing the effect of factorial additions of nitrogen and phosphorus on the prevalence of five different viruses (BYDV-MAV, BYDV-PAV, BYDV-SGV, BYDV-RMV, CYDV-RPV) in infected individuals of six grass hosts (Avena fatua, Bromus carinatus, Bromus hordeaceus, Elymus glaucus, Koeleria macrantha, and Taeniatherum caput-medusae. Permutations were constrained within unique block by site combinations testing for the effects of nitrogen, phosphorus, and host species after controlling for variation among blocks, sites, and states. Full model contained all two-way interactions between nitrogen, phosphorus, and host species. Note that among-host differences were not associated with host lifespan (annual or perennial), provenance (native or exotic), or phylogenetic group (bromes, oats, or rye). (DOCX) [file pone.0055675.s002.docx]

**Table S2.** Results of permutational multivariate analysis of variance (PERMANOVA) testing the effect of factorial additions of nitrogen and phosphorus on the prevalence of five different viruses (BYDV-MAV, BYDV-PAV, BYDV-SGV, BYDV-RMV, CYDV-RPV) in infected individuals of six grass hosts (*Avena fatua*, *Bromus carinatus*, *Bromus hordeaceus*, *Elymus glaucus* , *Koeleria macrantha*, and *Taeniatherum caput-medusae*. Permutations were constrained within unique block by site combinations testing for the effects of nitrogen, phosphorus, and host species after controlling for variation among blocks, sites, and states. Full model contained all two-way interactions between nitrogen, phosphorus, and host species. Note that among-host differences were not associated with host lifespan (annual or perennial), provenance (native or exotic), or phylogenetic group (bromes, oats, or rye).

| Source | D.F. | S.S | M.S. | F | p |
| --- | --- | --- | --- | --- | --- |
| Phosphorus | 1 | 0.378 | 0.378 | 2.094 | 0.023 |
| Host species | 5 | 1.191 | 0.238 | 1.321 | 0.017 |
| Residuals | 172 | 31.020 | 0.180 |  |  |
| Total | 178 | 32.589 |  |  |  |
