# Supplementary material for: Richness and Composition of Niche-Assembled Viral Pathogen Communities
Source: PLoS One. 2013 Feb 26;8(2):e55675. doi: 10.1371/journal.pone.0055675 (PMC3582609; doi:10.1371/journal.pone.0055675)
Supplement: Table S3 — Results of permutational multivariate analysis of variance (PERMANOVA) testng the effect of perennial grass cover, annual grass cover, forb cover, and factorial additions of nitrogen and phosphorus on the prevalence of five different viruses (BYDV-MAV, BYDV-PAV, BYDV-SGV, BYDV-RMV, CYDV-RPV) in infected individuals of six grass species (Avena fatua, Bromus carinatus, Bromus hordeaceus, Elymus glaucus, Koeleria macrantha, and Taeniatherum caput-medusae). Full model contained total live biomass, host species richness, perennial grass cover, annual grass cover, forb cover and all two-way interactions between. (DOCX) [file pone.0055675.s003.docx]

**Table S3.** Results of permutational multivariate analysis of variance (PERMANOVA) testing the effect of perennial grass cover, annual grass cover, forb cover, and factorial additions of nitrogen and phosphorus on the prevalence of five different viruses (BYDV-MAV, BYDV-PAV, BYDV-SGV, BYDV-RMV, CYDV-RPV) in infected individuals of six grass species (*Avena fatua*, *Bromus carinatus*, *Bromus hordeaceus*, *Elymus glaucus* , *Koeleria macrantha*, and *Taeniatherum caput-medusae*). Full model contained total live biomass, host species richness, perennial grass cover, annual grass cover, forb cover and all two-way interactions between nitrogen, phosphorus, and host species.

| Source | D.F. | S.S | M.S. | F | p |
| --- | --- | --- | --- | --- | --- |
| Live Biomass | 1 | 0.547 | 0.547 | 3.409 | 0.003 |
| Annual grass cover | 1 | 1.000 | 1.000 | 6.229 | 0.001 |
| Perennial grass cover | 1 | 2.456 | 2.456 | 15.308 | 0.001 |
| Forb cover | 1 | 0.418 | 0.418 | 2.606 | 0.024 |
| Phosphorus | 1 | 0.409 | 0.409 | 2.547 | 0.032 |
| Residuals | 173 | 27.759 | 0.160 |  |  |
| Total | 178 | 32.589 |  |  |  |
