# Supplementary material for: Richness and Composition of Niche-Assembled Viral Pathogen Communities
Source: PLoS One. 2013 Feb 26;8(2):e55675. doi: 10.1371/journal.pone.0055675 (PMC3582609; doi:10.1371/journal.pone.0055675)
Supplement: Table S4 — Linear coefficients for individual viral species from PERMANOVA (Table S2) testing the effect of factorial additions of nitrogen and phosphorus on the prevalence of five different viruses (BYDV-MAV, BYDV-PAV, BYDV-SGV, BYDV-RMV, CYDV-RPV) in infected individuals of six grass species (Avena fatua, Bromus carinatus, Bromus hordeaceus, Elymus glaucus, Koeleria macrantha, and Taeniatherum caput-medusae). (DOCX) [file pone.0055675.s004.docx]

**Table S4.** Linear coefficients for individual viral species from PERMANOVA (Table S2) testing the effect of factorial additions of nitrogen and phosphorus on the prevalence of five different viruses (BYDV-MAV, BYDV-PAV, BYDV-SGV, BYDV-RMV, CYDV-RPV) in infected individuals of six grass species (*Avena fatua*, *Bromus carinatus*, *Bromus hordeaceus*, *Elymus glaucus* , *Koeleria macrantha*, and *Taeniatherum caput-medusae*).

|  | MAV | PAV | RMV | RPV | SGV |
| --- | --- | --- | --- | --- | --- |
| Intercept | 0.0850 | 0.1059 | 0.1260 | 0.1166 | 0.1143 |
| Phosphorus addition | 0.0159 | 0.0300 | 0.0288 | -0.0130 | 0.0127 |
| Host (Bc versus Af) | 0.0083 | 0.0099 | -0.0393 | -0.0093 | 0.0336 |
| Host (Bh versus Af) | 0.0172 | 0.0226 | 0.0436 | 0.0223 | 0.0427 |
| Host (Eg versus Af) | -0.0210 | -0.0385 | 0.0047 | -0.0421 | -0.0234 |
| Host (Km versus Af) | 0.0282 | 0.0161 | 0.0013 | -0.0121 | -0.0243 |
| Host (Tc versus Af) | 0.0514 | 0.0338 | 0.0346 | 0.0323 | 0.0106 |
