# Supplementary material for: Richness and Composition of Niche-Assembled Viral Pathogen Communities
Source: PLoS One. 2013 Feb 26;8(2):e55675. doi: 10.1371/journal.pone.0055675 (PMC3582609; doi:10.1371/journal.pone.0055675)
Supplement: Table S5 — Linear coefficients for individual viral species from PERMANOVA (Table S4) testing the effect of perennial grass cover, annual grass cover, forb cover, and factorial additions of nitrogen and phosphorus on the prevalence of five different viruses (BYDV-MAV, BYDV-PAV, BYDV-SGV, BYDV-RMV, CYDV-RPV) in infected individuals of six grass species (Avena fatua, Bromus carinatus, Bromus hordeaceus, Elymus glaucus, Koeleria macrantha, and Taeniatherum caput-medusae). (DOCX) [file pone.0055675.s005.docx]

**Table S5.** Linear coefficients for individual viral species from PERMANOVA (Table S4) testing the effect of perennial grass cover, annual grass cover, forb cover, and factorial additions of nitrogen and phosphorus on the prevalence of five different viruses (BYDV-MAV, BYDV-PAV, BYDV-SGV, BYDV-RMV, CYDV-RPV) in infected individuals of six grass species (*Avena fatua*, *Bromus carinatus*, *Bromus hordeaceus*, *Elymus glaucus* , *Koeleria macrantha*, and *Taeniatherum caput-medusae*).

|  | MAV | PAV | RMV | RPV | SGV |
| --- | --- | --- | --- | --- | --- |
| Intercept | 0.0427 | 0.0364 | 0.1052 | 0.0659 | 0.1034 |
| Live Biomass | 0.0001 | 0.0002 | 0.0000 | 0.0002 | 0.0003 |
| Annual grass cover | -0.0003 | -0.0002 | 0.0005 | -0.0001 | -0.0005 |
| Perennial grass cover | 0.0039 | 0.0034 | 0.0000 | 0.0019 | 0.0025 |
| Forb (non-host) cover | 0.0000 | 0.0003 | -0.0003 | -0.0003 | -0.0013 |
| Phosphorus addition | 0.0114 | 0.0198 | 0.0202 | -0.0310 | -0.0068 |
