# Supplementary material for: Richness and Composition of Niche-Assembled Viral Pathogen Communities
Source: PLoS One. 2013 Feb 26;8(2):e55675. doi: 10.1371/journal.pone.0055675 (PMC3582609; doi:10.1371/journal.pone.0055675)
Supplement: Table S6 — Results of mixed-effects model testing the effect of perennial grass cover, annual grass cover, forb cover, and factorial additions of nitrogen and phosphorus on viral species richness (coinfection) by five different viruses (BYDV-MAV, BYDV-PAV, BYDV-SGV, BYDV-RMV, CYDV-RPV) in infected individuals of six grass hosts (Avena fatua, Bromus carinatus, Bromus hordeaceus, Elymus glaucus, Koeleria macrantha, and Taeniatherum caput-medusae. Full model contained total live biomass, host species richness, perennial grass cover, annual grass cover, forb cover and all two-way interactions between nitrogen, phosphorus, and host species. State, Site, Block, and Plot were treated as nested random effects. (DOCX) [file pone.0055675.s006.docx]

**Table S6.** Results of mixed-effects model testing the effect of perennial grass cover, annual grass cover, forb cover, and factorial additions of nitrogen and phosphorus on viral species richness (coinfection) by five different viruses (BYDV-MAV, BYDV-PAV, BYDV-SGV, BYDV-RMV, CYDV-RPV) in infected individuals of six grass hosts (*Avena fatua*, *Bromus carinatus*, *Bromus hordeaceus*, *Elymus glaucus* , *Koeleria macrantha*, and *Taeniatherum caput-medusae*. Full model contained total live biomass, host species richness, perennial grass cover, annual grass cover, forb cover and all two-way interactions between nitrogen, phosphorus, and host species. State, Site, Block, and Plot were treated as nested random effects.

|  | Value | Std.Error | DF | t-value | p-value |
| --- | --- | --- | --- | --- | --- |
| Intercept | 2.027 | 0.179 | 128 | 11.349 | 0.000 |
| Perennial grass cover | 0.016 | 0.006 | 23 | 2.920 | 0.008 |
